# Early Onset of Sex-Dependent Mitochondrial Deficits in the Cortex of 3xTg Alzheimer’s Mice

**DOI:** 10.3390/cells9061541

**Published:** 2020-06-24

**Authors:** Jelena Djordjevic, Subir Roy Chowdhury, Wanda M. Snow, Claudia Perez, Chris Cadonic, Paul Fernyhough, Benedict C. Albensi

**Affiliations:** 1 Division of Neurodegenerative Disorders, St Boniface Hospital Albrechtsen Research Centre, Winnipeg, MB R2H2A6, Canada; skr_chowdhury@yahoo.ca (S.R.C.); wsnow_2001@yahoo.ca (W.M.S.); cperez@sbrc.ca (C.P.); ccadonic@sbrc.ca (C.C.); pfernyhough@sbrc.ca (P.F.); 2 Department of Pharmacology & Therapeutics, University of Manitoba, Winnipeg, MB R2H2A6, Canada; 3 Research Institute in Oncology, CancerCare Manitoba, University of Manitoba, Winnipeg, MB R2H2A6, Canada

**Keywords:** Alzheimer’s disease, 3xTg, mitochondria, cortex, hippocampus

## Abstract

Alzheimer’s disease (AD) is a major public health concern worldwide. Advanced age and female sex are two of the most prominent risk factors for AD. AD is characterized by progressive neuronal loss, especially in the cortex and hippocampus, and mitochondrial dysfunction has been proposed to be an early event in the onset and progression of the disease. Our results showed early perturbations in mitochondrial function in 3xTg mouse brain, with the cortex being more susceptible to mitochondrial changes than the hippocampus. In the cortex of 3xTg females, decreased coupled and uncoupled respiration were evident early (at 2 months of age), while in males it appeared later at 6 months of age. We observed increased coupled respiration in the hippocampus of 2-month-old 3xTg females, but no changes were detected later in life. Changes in mitochondrial dynamics were indicated by decreased mitofusin (Mfn2) and increased dynamin related protein 1 (Drp1) (only in females) in the hippocampus and cortex of 3xTg mice. Our findings highlight the importance of controlling and accounting for sex, brain region, and age in studies examining brain bioenergetics using this common AD model in order to more accurately evaluate potential therapies and improve the sex-specific translatability of preclinical findings.

## 1. Introduction

Alzheimer’s disease (AD) is progressive, heterogeneous (multifactorial), and the most common age-related neurodegenerative disorder, with women being disproportionally affected [1,2]. It is characterized by loss of neurons and synapses in the cerebral cortex and hippocampus [3]. While plaques and tangles are hallmarks of the disease, a cascade of other pathological events, including synaptic dysfunction, oxidative stress, altered fatty acid, glucose and phospholipid metabolism, aberrant calcium homeostasis, neuroinflammation, as well as mitochondrial structural and functional changes, are consistently detected in men and women [4,5,6,7,8,9,10]. Mitochondrial anomalies occur early in disease progression [4], with individuals displaying early metabolic changes (impaired glucose metabolism, mitochondrial dysfunction, and altered energy homeostasis) prior to the emergence of any histopathological or clinical abnormalities. Our previous studies showed early changes in mitochondrial membrane potential and oxygen consumption rates in the cortex [11], and impaired brain metabolic activity in adult 3xTg male mice, as measured with FDG-PET [12]. Importantly, these bioenergetic changes were found in the absence of excess amyloid-β (Aβ) plaque deposition in the brain, a neuropathological hallmark of the disease, suggesting that brain hypometabolism occurs prior to AD pathology. A recent study by Jadiya et al. (2019) also showed that in the 3xTg mouse model impaired mitochondrial Ca^2+^ (mCa^2+^) efflux capacity precedes neuropathology [13].

The brain is one of the most energy-expensive organs in the body and is critically dependent on oxidative phosphorylation as a main source of energy. It consumes 20% of all inspired oxygen and 25% of the body’s energy stores, although it accounts for only 2% of total body weight [14]. Mitochondria are critical organelles required for neurons to execute the complex processes of neurotransmission and plasticity, and to establish membrane excitability. As a consequence, mitochondrial dysfunction can result in disrupted morphological and functional responses to synaptic activity, maintenance and restoration of membrane potential, neurotransmitter packaging and release, calcium homeostasis, increased reactive oxygen species (ROS) generation, including H_2_O_2_ production, and decreased oxygen uptake and ATP production, all of which are known pathological hallmarks of AD [15].

To ensure the maintenance of a healthy mitochondrial population, cells are equipped with mitochondrial quality-control systems to regulate mitochondrial shape, function, and mass. Mitochondrial networking, with other organelles and with each other, occurs in part via dynamic fusion and fission processes [16]. The protein machinery regulating mitochondrial fission and fusion is now well characterized. Processes of fission and fusion have a different impact on mitochondrial physiology: mitochondrial fission is required for the selective elimination of depolarized mitochondria [17] and occurs during apoptosis [18], but it also allows mitochondrial renewal, redistribution, and proliferation into synapses, thus maintaining a pool of healthy mitochondria, whereas fusion is required for mtDNA maintenance, probably because it allows mtDNA exchange between mitochondria, and it facilitates mitochondrial movement and distribution across axons and synapses, suggesting a protective mechanism helping the maintenance of sufficient bioenergetic levels adjusted to situations with high-energy demands. Clinical studies have found a link between mitofusin 2 gene polymorphisms and individual susceptibility to late-onset AD [19], and the role of Drp1 in AD pathogenesis has also been well documented [20]. Abnormal mitochondrial dynamics (increased fission and decreased fusion) is well documented in AD [21] and other neurodegenerative diseases, and poor mitochondrial distribution in the axons of AD patients is also reported [22]. 

Current paradigms of AD also implicate oxidative stress-mediated neuronal damage as a contributing factor to the early pathology, with mitochondrial membrane potential regulating ROS production. The inner mitochondrial membrane anion transporters called the uncoupling proteins (UCPs) function as regulators of cellular homeostasis by mitigating oxidative stress. They facilitate the transmembrane transfer of protons and are important regulators of energy fluxes, oxidative stress, and intra-cellular calcium homeostasis. The main UCP isoforms expressed in the brain are UCP2, UCP4, and UCP5 [23]. We examined the role of UCP4, since genetic variability of the UCPs in humans has been shown to be associated with longevity [24], and genetic variability of UCP4 in particular with individual susceptibility to late-onset AD [25]. It is prominently expressed in neurons from hippocampus, cortex, substantia nigra, pars compacta, striatum and cerebellum and, at lower levels, in astrocytes, and appears to have a critical role in helping neurons to cope with conditions of metabolic and oxidative stress [26].

Great progress has been made in AD research over the past decade, but limited attention has been given to sex and gender differences in AD, leaving significant knowledge gaps in research and a lack of awareness among the research community on the importance of sex and gender. In the 3xTg model of AD, sex differences have consistently been reported, including a significantly greater Aβ burden [27,28] and larger behavioral deficits [29] in female 3xTg mice than age-matched males. Studies evaluating the impact of sex on mitochondrial morphology and function in models of AD, however, are very limited.

In this study, we used the 3xTg mouse model that exhibits age-related increases in Aβ production and plaque deposition as well as tau pathology to examine how mutations associated with familial AD affect mitochondrial function as a function of sex. We further studied sex differences in mitochondrial deficits specifically in the cortex and hippocampus, two brain regions that demonstrate robust AD-related pathology in this model [30], during the aging process, since prior studies have reported that some genes increase the risk and progression of AD in one sex, but not the other [31,32]. Mitochondrial bioenergetic profiles, markers of morphological changes (Drp1 and Mfn2), and the levels of mitochondrial uncoupling protein UCP4 were evaluated in both males and females in 3xTg mice, as few studies thus far have addressed brain-region and sex-specific differences for these proteins in existing AD mouse models.

## 2. Materials and Methods

### 2.1. Animals

The triple-transgenic (3xTg) mouse strain is a widely used and valuable model to study AD-related molecular mechanisms because it develops both Aβ and tau brain pathology [30,33]. It possesses the M146V mutation knocked into the PS1 gene and overexpresses human APPswe and tauP301L16, with translation of these overexpressed transgenes appearing restricted to the central nervous system, including the hippocampus and cerebral cortex. This strain exhibits age-dependent neuroanatomical and cognitive parallels to AD, including Aβ plaque formation, neurofibrillary tangles, and memory deficits, making it relevant to AD in humans. Intracellular Aβ accumulation is detected in some brain regions as early as 3–4 months of age. Extracellular Aβ deposits appear by six months in the frontal cortex and become more extensive by twelve months. Synaptic transmission and long-term potentiation (LTP) are impaired by 6 months, and hyperphosphorylated tau is detected at 12–15 months [30]. The C57BL/6 mouse was used as a background control strain since our 3xTg mice were maintained on C57BL/6 for 8 generations. All mice were housed in individual cages in a Canadian Council of Animal Care (CCAC)-accredited vivarium with twelve-hour light/dark cycles (i.e. light from 7:00 a.m. to 7:00 p.m.). Food and water were made available to the mice ad libitum.

To assess age-related sex differences in mitochondrial deficits in 3xTg mice, male and female 3xTg mice were divided into 6 groups according to sex and age (*n* = 4–6 per group). Groups of both males and females were killed at 2 months, 6 months, and 14 months of age. All mice in this study were killed using rapid decapitation following loss of consciousness produced by a gas chamber treated with 95% isoflurane gas. The cortex and hippocampus were dissected out from the brain immediately following decapitation. All animal experiments in this study were conducted according to protocols approved by the University of Manitoba—Animal Office of Research Ethics and Compliance and Review Committee and in full compliance with the Canadian Council on Animal Care (Protocol Reference Numbers: 17-020/1, valid from 7 July 2018 to 6 July 2019, and 17-020/2, valid from 7 July 2019 to 6 July 2020, AC11275).

### 2.2. Preparation of Isolated Mitochondria from Cortical and Hippocampal Tissues

Whole tissue homogenates of cortical and hippocampal tissue were prepared in a glass homogenizer containing 1 mL of mitochondrial isolation buffer (70 mM sucrose, 210 mM mannitol, 5 mM HEPES, 1 mM EGTA, 0.5% BSA). The tissue was homogenized with ten strokes each from pestle A, then pestle B, and the resultant homogenate was centrifuged at 800× *g* for 10 min at 4 °C. The resulting supernatants were collected and centrifuged at 8000× *g* for 15 min at 4 °C. The new supernatants were discarded, and the pellets were saved. The pellets were washed in mitochondria isolation buffer and centrifuged at 8000× *g* for 15 min one more time at 4 °C. The final supernatant was discarded, and the final pellet (isolated mitochondrial fraction) was resuspended in 100 μL of mitochondrial isolation buffer. A small volume of the suspension was collected for use in a colorimetric protein assay (Bio-Rad DC Protein Assay kit) to determine the concentration of total protein. Protein concentrations of the samples were measured using light absorbance at 750 nm in a microplate reader.

### 2.3. Measurement of Mitochondrial Respiration Rates in Cortex and Hippocampus

Complex-I-dependent mitochondrial respiration was assessed by measuring oxygen consumption rate (OCR) in real time [34], in freshly isolated mitochondria from the cortex and hippocampus, using the Seahorse XF24 Analyzer (Agilent Technologies, CA). Twenty micrograms of freshly isolated mitochondrial protein were diluted in mitochondrial assay solution (MAS, volume of 50 μL) containing 70 mM sucrose, 220 mM mannitol, 10 mM KH2PO4, 5 mM MgCl2, 5 mM HEPES, 1 mM EGTA, and 0.2% BSA (pH 7.2), and plated in each well of the V7 culture plate. The plate was then centrifuged for 20 min at 2000 rpm, at 4 °C. After centrifugation, 400 μL of MAS with pyruvate (10 mM) and malate (2 mM) was added to each well, and the plate was incubated at 37 °C for 8–10 min. Basal level of oxygen consumption was measured in the presence of Complex I substrates, pyruvate and malate. Adenosine diphosphate (ADP, 2 mM), oligomycin (1 μM), carbonylcyanide p-trifluoromethoxyphenylhydrazone (FCCP, 4 μM) and rotenone (1 μM) + antimycin A (1 μM) were injected consecutively through ports A, B, C and D in the Seahorse Flux Pak cartridges, to determine coupled respiration, uncoupled respiration, and non-mitochondrial oxygen consumption [35]. Coupled respiration that drives oxidative phosphorylation of ADP to ATP was measured after the addition of ADP. Oligomycin was then added to terminate coupled respiration through inhibition of ATP synthase. The protonophore FCCP was added to stimulate uncoupling of the respiratory chain and allow for the measurement of uncoupled respiration. Finally, injection of rotenone (Complex I inhibitor) and antimycin (Complex III inhibitor) blocked the flux of electrons through these complexes so that no oxygen was further consumed at cytochrome c oxidase (non-mitochondrial respiration rates). OCR data were calculated with subtraction of non-mitochondrial respiration rates. 

### 2.4. Western Blot Analysis

Bio-Rad TGX Stain Free™ Acrylamide kit was used for all Western blot (WB) procedures. The samples were prepared with denaturing buffer according to Laemmli [36], boiled for 10 min at 55 °C, and subjected to electrophoresis on 10% sodium dodecyl sulfate-polyacrylamide gel (SDS-PAG). Subsequently, the gels were activated using a Bio-Rad UV transilluminator (ChemiDoc™ MP Imaging System). Wet-transfer system was used to transfer proteins to nitrocellulose membrane (Bio-Rad, 0.2 μm). Following the wet transfer, total protein was detected by the ChemiDoc™ MP imager. Membranes were blocked for 1 h at room temperature in 1× Tris-buffered saline with 0.1% Tween-20 (TBS-T) containing 5% milk. Thereafter, membranes were incubated overnight, with selected primary antibodies in TBS-T with 5% milk at 4 °C. The following day, the membranes were washed in TBS-T (three ten-minute washes) and incubated with selected secondary antibody, also prepared in 5% milk in TBS-T, for 2 h at 4 °C. Antibody binding was detected with enhanced chemiluminescence (ECL) solution (Bio-Rad Clarity™ Western ECL Substrate Kit) and imaged with the ChemiDoc™ MP imager (Bio-Rad). PageRuler™ Plus Protein Ladder was used to determine the molecular weights of the protein bands. The following primary antibodies were used: Total OXPHOS Rodent WB Antibody Cocktail (ab110413, Abcam), VDAC1/Porin (ab14734, Abcam), UCP4 (N-16) (sc-17582, Santa Cruz Biotechnology Inc.), Anti-Drp1 antibody (4F6) (ab156951, Abcam), and Anti-Mitofusin 2 antibody (ab56889, Abcam). The secondary antibody Peroxidase AffiniPure Goat Anti-Mouse IgG (Jackson Immuno Research) was used for OXPHOS, Porin, Drp1, and Mfn2, and Rabbit Anti-Goat IgG (Invitrogen) was used for UCP4.

### 2.5. Statistical Analysis

Two-tailed Student’s t-test was used for bioenergetics parameters, to determine between-group differences. Two-way ANOVA was used for analysis of WB data, to determine the effects of genotype, sex, and their potential interactions (2 × 2). In cases of a significant interaction effect, simple main effect was calculated using a t-test. All values were considered statistically significant if the *p*-value was less than 0.05.

## 3. Results

### 3.1. Bioenergetic Profiles in Isolated Mitochondria from Cortex and Hippocampus of 3xTg Mice at Different Ages

**Figure 1:**
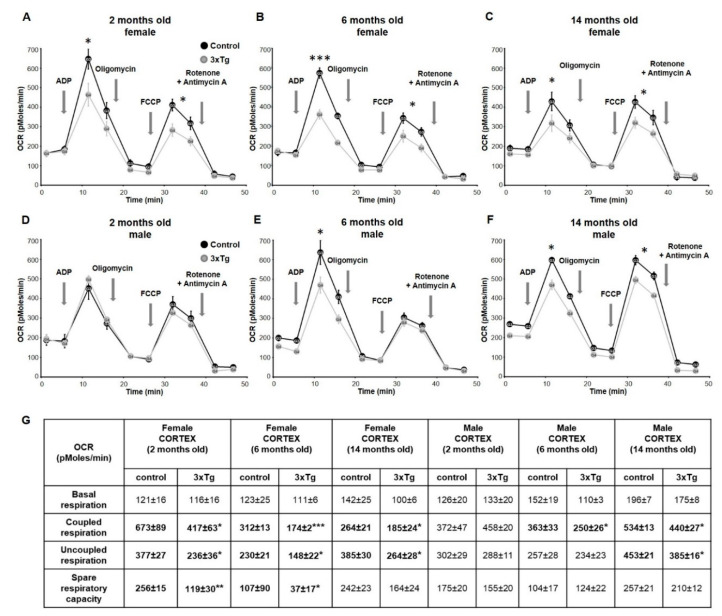
Representative measurements of oxygen consumption rates in mitochondria isolated from the cortex of 3xTg and age-matched control females at 2 months (**A**), 6 months (**B**) and 14 months of age (**C**), and males (**D**–**F**). Basal level of OCR was measured in the presence of Complex I substrates pyruvate and malate. Coupled respiration was measured after the addition of ADP (2 mM). Oligomycin (1 μM) was then added to terminate coupled respiration through inhibition of ATP synthase. The uncoupled respiration rate was determined by adding the uncoupling agent FCCP (4 μM) to remove the pH gradient and enable maximal rates of electron transport. The injection of rotenone (1 μM) and antimycin A (1 μM) blocked respiratory electron flux at complexes I and III (non-mitochondrial respiration rates). Calculated coupled respiration at state 3 and uncoupled respiration were significantly decreased (* *p* < 0.05) in cortical mitochondria of 3xTg female mice at 2, 6 and 14 months, and SRC was significantly reduced at 2 and 6 months (Table **G**, in bold). In 3xTg males, coupled respiration was significantly decreased at 6 and 14 months, and uncoupled respiration was down at 14 months (* *p* < 0.05). Data are expressed as OCR in pmoles/min, and values are expressed as mean ± SEM of *n* = 4–6 replicates.

In order to evaluate the functionality of the respiratory chain, we measured Complex I-dependent respiration in state 3 (using the substrates pyruvate and malate) and maximal respiration (attained by adding the uncoupler FCCP). State 3 respiration measures the capacity of the mitochondria to metabolize oxygen and the selected substrate in the presence of a defined amount of ADP, which is a substrate for the ATP synthase (Complex V). Measurements are shown of OCR in freshly isolated cortical mitochondria from 2, 6, and 14-month-old females (Figure 1A–C) and male 3xTg mice (Figure 1D–F) and their respective controls. We observed a significantly reduced state 3 respiration (with pyruvate/malate) as well as a significant reduction in uncoupled respiration in the cortex of 3xTg females at 2 months of age. Calculation of the spare respiratory capacity (SRC) (Figure 1G, * *p* < 0.05) also showed a significant decrease, indicating that the ability of the cortex to respond to increased energy demand is impaired in young 3xTg females. Spare respiratory capacity has been shown to correlate with enhanced cell survival [37] and conversely, reduced SRC has been associated with neuronal cell death and disease [38]. In males, mitochondrial deficits did not appear until 6 months of age, when coupled respiration was significantly down (* *p* < 0.05), and also 14 months, when both coupled and uncoupled respiration were down as compared to age-matched controls (Figure 1G). 

**Figure 2:**
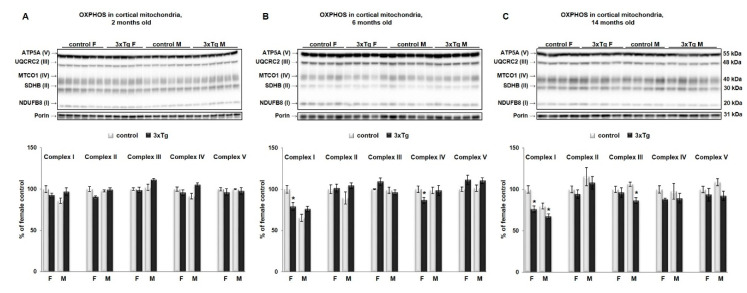
Relative levels of OXPHOS proteins in cortical mitochondria of 3xTg mice and their respective controls. Representative WBs (upper panels) for Complex I (NADH dehydrogenase beta subcomplex subunit 8, NDUFB8), Complex II (succinate dehydrogenase subunit B, SDHB), Complex III (cytochrome b-c1 complex subunit 2, UQCRC2), Complex IV (Cytochrome c oxidase subunit 1, MTCO1), and Complex V (ATP synthase subunit alpha, ATP5A) and relative quantification (lower panels) for protein levels of OXPHOS subunits normalized to porin, in 3xTg female and male mice and their respective controls at 2 months (**A**), 6 months (**B**) and 14 months of age (**C**). Results are expressed as percent change from the control mean of 100% ± SEM of *n* = 4–6 animals per group (* *p* < 0.05). Contrary to our findings in the cortex, hippocampal mitochondria showed increased coupled respiration in female 3xTg mice at 2 months of age (Figure 3A,D; * *p* < 0.05), followed by the increase in protein level of Complex I and IV subunits (Figure 3E, * *p* < 0.05). This increase was not sustained at later stages, as we found no changes in OCR or protein level of Complex I–V subunits in older 3xTg female mice (Figure 3B–D). Neither mitochondrial bioenergetics’ parameters nor protein levels of analyzed subunits of Complex I–V in the hippocampus of 3xTg males were altered in any of the studied age groups.

Interestingly, WB results showed that decreased protein levels of both NADH dehydrogenase beta subcomplex subunit 8 of Complex I and MT-CO1 subunit of Complex IV in cortical mitochondria from 3xTg mice (* *p* < 0.05) appeared later than functional deficits as such differences were not detected until 6 months of age (Figure 2B) in females and 14 months of age in males (Figure 2C, only Complex I was significantly decreased, * *p* < 0.05). No statistically significant differences were detected in protein levels of any of the OXPHOS subunits at 2 months of age (Figure 2A).

### 3.2. Changes in Mitochondrial Morphology in 3xTg Mouse Brain

**Figure 4:**
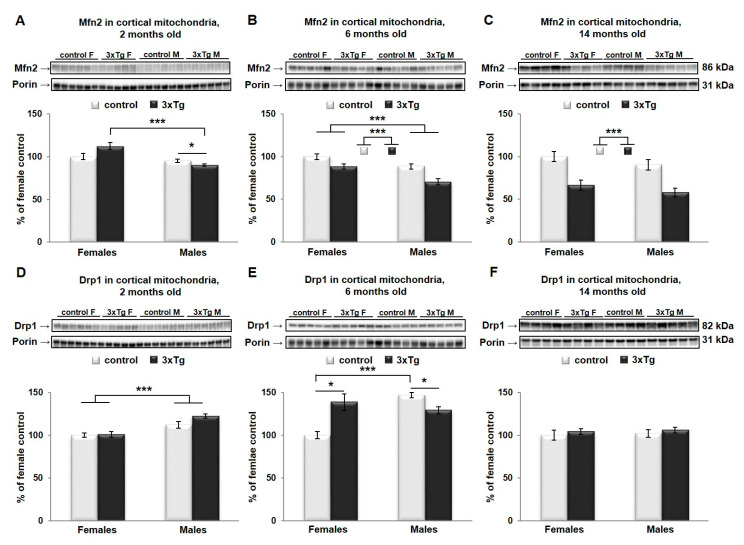
Mfn2 and Drp1 protein levels in cortical mitochondria. Representative WB for Mfn2 (**A**–**C**) and Drp1 (**D**–**F**) and their relative quantification normalized to porin protein levels in the cortex of 2-, 6- and 14-month-old 3xTg female and male mice and their respective controls. Data were analyzed by two-way ANOVA, and results are expressed as percent change from control mean of 100% ± SEM of *n* = 4–6 animals per group and Mfn2 protein levels were significantly different in cortical mitochondria as a function of sex (*** *p* < 0.001) in 2- and 6-month-old mice and genotype (*** *p* < 0.001) in 6- and 14-month-old mice, with a significant interaction effect between sex and genotype only in 2-month-old mice (** *p* < 0.01). Analysis of simple main effects showed significantly lower levels of Mfn2 in 3xTg males versus 3xTg females (*** *p* < 0.001) and downregulation in the 3xTg male mice compared to controls (*p* = 0.03). Drp1 protein levels were significantly different in cortical mitochondria as a function of sex in 2- and 6-month-old mice (*** *p* < 0.001 and ** *p* < 0.01 respectively), with a significant interaction effect between sex and genotype only in 6-month-old mice (*** *p* < 0.001). Here, levels of Drp1 were significantly higher in males than females in the C57BL/6 mouse (*** *p* < 0.001) and in the 3xTg female relative to their background control females (* *p* < 0.05). Drp1 was significantly reduced in 3xTg males relative to male control mice (* *p* < 0.05).

As suggested earlier, mitochondrial size and shape as regulated by mitochondrial dynamics are very important for their proper function. In cortical mitochondria, two-way ANOVA indicated that Mfn2 protein levels were significantly different as a function of sex (Mfn2 protein levels were higher in females, *** *p* < 0.001) in 2- and 6-month-old mice (Figure 4A,B) and as a function of genotype (Mfn2 protein levels were lower in 3xTg animals, *** *p* < 0.001) in 6- and 14-month-old mice (Figure 4B,C), with a significant interaction effect between sex and genotype only at 2 months old (** *p* < 0.01). Analysis of simple main effects in this group indicated a sex effect in 3xTg mice only, with significantly lower levels of Mfn2 in males versus females (*p* < 0.001). The effect of genotype was significant in male mice only, with a downregulation in the 3xTg model (*p* = 0.03). Drp1 protein levels were significantly different in cortical mitochondria as a function of sex in 2- and 6-month-old mice (Drp1 protein levels were higher in males, *** *p* < 0.001 and ** *p* < 0.01 respectively, Figure 4D,F), with a significant interaction effect between sex and genotype only in 6-month-old mice (*** *p* < 0.001). Here, levels of Drp1 were significantly higher in males than females in C57BL/6 mice (*** *p* < 0.001), with no such effect of sex in 3xTg mice. Drp1 levels were significantly higher in the 3xTg female relative to control females (*p* = 0.013) but were reduced in 3xTg males relative to male control mice (*p* = 0.014).

**Figure 5:**
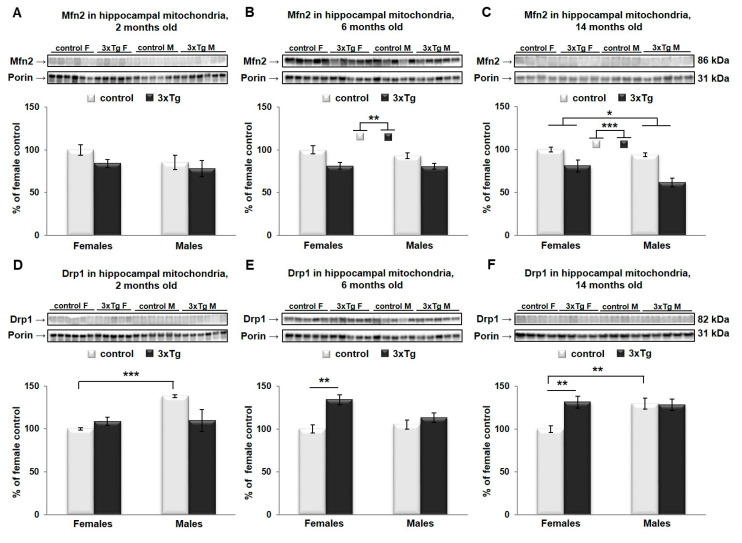
Mfn2 and Drp1 protein levels in hippocampal mitochondria. Representative WBs for Mfn2 (**A**–**C**) and Drp1 (**D**–**F**) and their relative quantification normalized to porin protein levels in the hippocampus of 2-, 6- and 14-month-old 3xTg female mice and their respective controls. Data were analyzed by two-way ANOVA, and results are expressed as percent change from control mean of 100% ± SEM of *n* = 4–6 animals per group. Mfn2 protein levels were significantly different in hippocampal mitochondria as a function of genotype in 6- and 14-month-old mice (** *p* < 0.01 and *** *p* < 0.001 respectively) and sex (* *p* < 0.05) in 14-month-old mice, with no significant interaction effect between sex and genotype in any group. Drp1 protein levels were significantly different as a function of sex in two 2-month-old mice (* *p* < 0.05), and as a function of genotype in 6- and 14-month-old mice (** *p* < 0.01 and * *p* < 0.05 respectively), with a significant interaction effect (* *p* < 0.05) between sex and genotype in all three groups. At 2 months, analysis of simple main effects showed significantly higher Drp1 levels in control males than in females (*** *p* < 0.001). Drp1 levels were significantly higher as a function of genotype in females only in both age groups (6-month: ** *p* = 0.002; 14-month: ** *p* = 0.007). The effect of sex was significant only in C57BL/6 control mice at 14 months, where levels were elevated in males versus females (** *p* < 0.01).

In the hippocampal mitochondria, two-way ANOVA analysis revealed that Mfn2 protein levels were significantly different as a function of genotype in 6- and 14-month-old mice (Mfn2 protein levels were lower in 3xTg animals, ** *p* < 0.01 and *** *p* < 0.001 respectively, Figure 5B,C), and as a function of sex in 14-month-old mice (Mfn2 protein levels were higher in females, * *p* < 0.05, Figure 5C). No significant interaction effect between sex and genotype was detected for Mfn2 at any age group. At 2 months, there was a significant interaction effect for Drp1; analysis of simple main effects indicated an effect of sex in C57 mice only, with significantly higher levels in males than in females (*** *p* < 0.001). Drp1 protein levels differed significantly as a function of genotype in 6- and 14-month-old mice (** *p* < 0.01 and * *p* < 0.05 respectively), with a significant interaction effect (* *p* < 0.05) between sex and genotype in these two age groups as well. Drp1 levels were significantly higher as a function of genotype in females only in both age groups (6-month: *p* = 0.002; 14-month: *p* = 0.007). The effect of sex was significantly only in C57BL/6 mice at 14 months, where levels were elevated in males versus females (*p* < 0.01; Figure 5E,F).

### 3.3. Uncoupling Protein 4 in Mitochondria of 3xTg Mouse Brain

**Figure 6:**
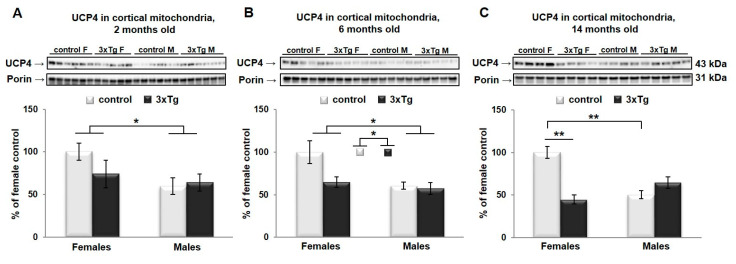
UCP4 protein levels in cortical mitochondria. Representative WBs for UCP4 and their relative quantification normalized to porin protein levels in the cortical mitochondria from 3xTg female and male mice at 2 (**A**), 6 (**B**) and 14 months of age (**C**). Data were analyzed by two-way ANOVA, and results are expressed as percent change from control mean of 100% ± SEM of *n* = 4–6 animals per group. UCP4 protein levels were significantly different as a function of sex (* *p* < 0.05), regardless of age, and of genotype at 6 and 14 months (* *p* < 0.05 and ** *p* < 0.01 respectively), with a significant interaction effect between sex and genotype at 14 months (* *p* < 0.05). Simple main effects in this age group revealed that control males had significantly less UCP4 than females (** *p* = 0.001) and that 3xTg females had significantly less relative to C57BL/6 females (** *p* = 0.001).

Levels of UCP4 protein were also analyzed by two-way ANOVA. There was a significant main effect of sex in UCP4 protein levels in cortical mitochondria (UCP4 protein levels were higher in females, * *p* < 0.05, Figure 6A–C), regardless of age. UCP4 protein levels were significantly different as a function of genotype at 6 and 14 months (UCP4 protein levels were lower in 3xTg animals, * *p* < 0.05 and ** *p* < 0.01 respectively, Figure 6B,C), with a significant interaction effect between sex and genotype at 14 months (* *p* < 0.05). Simple main effects in this age group revealed that in control mice, males had significantly less UCP4 than females (** *p* = 0.001), with no such sex effect in 3xTg mice. The effect of genotype was significantly only in females, with a reduction in 3xTg mice relative to C57BL/6 control mice (** *p* = 0.001). 

**Figure 7:**
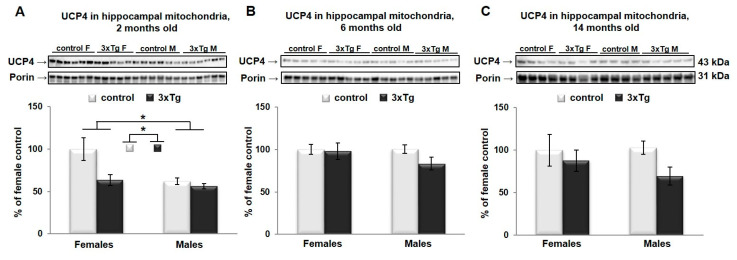
UCP4 protein levels in hippocampal mitochondria. Representative WBs for UCP4 and their relative quantification normalized to porin protein levels in the hippocampal mitochondria from 3xTg female and male mice at 2 (**A**), 6 (**B**) and 14 months of age (**C**). Data were analyzed by two-way ANOVA, and results are expressed as percent change from control mean of 100% ± SEM of *n* = 4–6 animals per group. UCP4 protein levels were significantly different as a function of sex (* *p* < 0.05) and as a function of genotype (* *p* < 0.05) only at 2 months, with a trend towards a significant interaction effect between sex and genotype (*p* = 0.067).

In hippocampal mitochondria, two-way ANOVA analysis revealed that UCP4 protein levels were significantly different as a function of sex (UCP4 protein levels were higher in females, * *p* < 0.05, Figure 7A) and as a function of genotype (UCP4 protein levels were lower in 3xTg animals, * *p* < 0.05, Figure 7A) only at 2 months, with a trend towards a significant interaction effect between sex and genotype (*p* = 0.067).

## 4. Discussion

The complexity of the etiological factors and different pathological manifestations of AD make it difficult to clinically define the most important and/or earliest contributor to the onset and progression of the disease if such a singularly important factor exists. Current evidence strongly supports the idea that mitochondrial dysfunction is an early important event in the onset and progression of AD and is speculated to drive subsequent synaptic dysfunction and neuronal loss. Further, the incidence of the disease is higher in women than in men, which warrants more research on sex-specific differences in the development of the disease.

In the present study, the aim was to investigate early-onset and progression of mitochondrial dysfunctions in the 3xTg mouse that over-expresses mutant APP, tau, and PS1 by evaluating mitochondrial bioenergetic profiles in hippocampus and cortex in both males and females at different ages. We further measured accepted markers of mitochondrial dynamics, Drp1 and Mfn2, known to affect mitochondrial function, and often altered in AD. Our results provide evidence that mitochondrial functional and dynamics changes exhibit different rates of alteration depending on sex, brain region, and age-related disease progression.

One of the main findings of our study was that the cortex appeared to be more susceptible to changes in mitochondrial bioenergetics than the hippocampus in the 3xTg mouse. We further confirm that metabolic mitochondrial deficits in the cortex occur early and precede the typical development of AD pathology reported in this strain [39], by a substantial amount of time, particularly in females. Lower rates of OCR were evident very early in the cortex of 3xTg females (at 2 months), while in males, a decline in mitochondrial function was not detected until later at 6 months (* *p* < 0.05). What was interesting in our study was that a decline in mitochondrial function preceded a reduction in protein levels of Complex I and IV subunits. Lower protein levels of Complex I and Complex IV in the female cortex were not statistically significant until 6 months of age, and in males, protein levels of Complex I subunit were down at 14 months. Complex I and Complex IV deficiencies occurred in the absence of changes in other OXPHOS proteins. Similar observations have been reported in PD (Parkinson’s disease) patients, where the majority of substantia nigra neurons showed either Complex I, or combined Complex I and Complex IV deficiencies. It was suggested that Complex IV deficiency in nigral neurons only occurs in the presence of Complex I deficiency and that this molecular signature may arise from mtDNA alterations [40].

Early mitochondrial deficits (at 3 months of age) have previously been reported in the whole brain from female Thy-1 APP mice, which also exhibit Aβ overproduction [41] and 3xTg mice [42]. However, these studies did not examine males. Manczak et al. [43] found similar results in the frontal cortex of AD patients, with decreased expression of Complex I subunit, but increased Complex IV subunit. Interestingly, they reported a decrease in Complex I subunit 1, which is mitochondrially encoded, attributing it to potential frequent mutations in mtDNA. Here, we report a decrease in Complex I subunit 8, which is nuclear encoded. Studies have shown that genes that encode protein subunits that participate in the same OXPHOS complexes tend to co-express [44,45,46]. Studies on rat brain synaptosomes showed that Complex I is rate-limiting for OCR, suggesting that Complex I deficiencies that occur in neurodegenerative disorders, such as AD and PD, are sufficient to compromise ATP production and mitochondrial respiration rates within synaptic regions and thus contribute to synaptic impairments seen in these diseases [47]. Studies have shown that ROS formation is particularly high when Complex I is inhibited [48], and high ROS levels are one of the hallmarks of AD [49]. In addition to the well-established Complex I deficiency in AD, Complex IV dysfunction has also previously been implicated in the pathogenesis of AD. Interestingly, the primary risk factor for AD is aging, which itself is correlated with the presence of Complex IV-deficient neurons, suggesting that age-related damage accumulation could contribute to neuronal demise [50].

The decrease in energy metabolism in cortical mitochondria found in 2 month-old 3xTg females reported here contradicts the idea that hormonal deficiencies in the post-menopausal period is the main factor for the greater risk of AD in females and the drop in energy metabolism [51]. Such early mitochondrial deficits could be explained by the intracellular accumulation of Aβ and Aβ-induced toxic events that occur much earlier than the formation of senile plaques [52]. In our study, a decrease in Complex I and IV protein levels was evident in females at the age of 6 months but not in males. Clinical data from Valla et al. also show a reduction of cytochrome c oxidase in the posterior cingulate cortex, and this reduction appeared to be greater in women [53]. Furthermore, a clinical study by Bokde et al. showed that cerebral hypometabolism is not simply caused by brain atrophy [54], or a faster rate of cognitive and functional decline [55], but is actually a consequence of true loss of functional activity. Wenfeng et al. (2014) performed an assessment of oxidative stress in the course of brain aging in CRND8 mice that develops early and robust Aβ plaque deposition and found that ROS production was significantly higher in the cortex (but not hippocampus) as early as 2 months of age [56]. The study by Carroll et al. (2010) showed an age-related increase in Aβ load in 3xTg mice starting at 6–8 months, with a consistently higher Aβ burden in female 3xTg mice [28]. This study also showed that the effect of sex on Aβ load was most noticeable in the frontal cortex. Consistent with findings in Aβ, others have also noted elevated levels of hippocampal neurofibrillary tangles in females relative to males at 12 months when detected immunohistochemically [57] Prior to this age, neurofibrillary tangle pathology is limited in this region [30]. Reports from the AD neuroimaging initiative (ADNI) showed that annual cortical atrophy rates were higher in women than in men and are correlated with Aβ and tau changes in cerebrospinal fluid and with ApoE4 allele status [58]. Other studies also showed that female AD patients have more cortical atrophy than male AD patients [59]. Vandal et al. [27] found that glucose intolerance and cortical amyloid pathology worsened with age in 3xTg mice, particularly in females. 

Hippocampal mitochondria showed increased coupled, state 3 respiration when metabolizing the site I substrates, pyruvate and malate, but only in 2-month old 3xTg females, with no changes detected in older age groups. This was followed by statistically significant increases in protein levels of Complex I and IV subunits. The increases might reflect a compensatory or initially protective response of affected cells. As stated before, we didn’t detect any changes in the hippocampus in 3xTg males in either mitochondrial bioenergetics parameters or protein levels of analyzed subunits of Complex I–V. This result is, to some extent, in alliance with our previous findings in CRND8 mice [60] where we also showed no differences in mitochondrial function in the hippocampus of 3xTg males, whereas in females, an increase was observed at 14 months of age. Similar results for females were reported in 3xTg mice in the study by Coskun et al. [61] where females showed a striking biphasic response with an initially elevated brain state 3 respiration rate followed by progressive decline. By contrast, the male 3xTg mice in their study show a consistently reduced forebrain mitochondrial respiration rate, both when coupled and uncoupled to ATP synthesis.

Proper function of mitochondria is closely related to its morphological properties, which are regulated by the dynamic processes of fission and fusion, and the balance between fusion and fission determines the fate of defective mitochondria. It is also well documented that Aβ plays a role in mitochondrial network collapse, and increased production of Aβ and the interaction of Aβ with Drp1 are crucial factors in mitochondrial fragmentation, abnormal mitochondrial dynamics, synaptic loss and neuronal damage in AD-affected neurons [62,63,64,65]. 

Our results showed that at the earlier ages (2 and 6 months), there is a sex-based difference in the balance between fusion and fission proteins in the cortex, regardless of the pathology. The dynamic balance of fission and fusion is greatly shifted toward fission in the male cortex, since they appear to have overall higher levels of Drp1 protein and lower levels of Mfn2 protein, compared to females. One explanation could be that female mitochondria are more differentiated than male mitochondria [66]. Our results also showed decreases in Mfn2 protein level in the cortical mitochondria from both female and male 3xTg (6 months), which coincided with an increased level of Drp1. This shift in balance toward fission, in affected brain regions of 3xTg mice could compromise their ability to meet metabolic demands, leading to an increased susceptibility to cell death and apoptosis. Our results are consistent with the findings from other labs using AD transgenic models [67] or primary neurons from AD transgenic mice [68]. Clinical studies also showed decreased Mfn2 and increased Drp1 gene expression in the cortex of AD patients [62]. Mitofusin 2 is essential for mitochondrial morphology and function as well as maintenance of mitochondrial coenzyme Q levels. Loss of Mfn2 results in mitochondrial heterogeneity and dysfunction. It can impair the mitochondrial respiratory chain capacity even without affecting the levels of OXPHOS components. Studies on the hiPSCs-derived cortical neurons showed that knocking down Mfn2 compromises mitochondrial metabolism and network, neurogenesis and synapse formation [69]. On the other hand, an increase in Drp1 is related to abnormal mitochondrial distribution, since this protein is critical for mitochondrial division, size, shape, and distribution throughout the neuron, from cell body to axons, dendrites, and nerve terminals.

The process of oxidative phosphorylation is coupled to production of ROS, which in excess can have a deleterious effect on the cell. The role of oxidative stress and excessive ROS has been well documented in the pathogenesis AD. An integral part of mitochondrial physiology is the uncoupling of respiration from oxidative phosphorylation, a process mediated by mitochondrial UCPs. The UCPs are a group of proteins with neuroprotective properties, and their induction in specific brain regions has been suggested to help neurons cope with metabolic and oxidative stress. The isoform UCP4 is expressed predominantly in the central nervous system, and the amount of protein varies between different brain regions, with the highest protein content found in the cortex [26]. What was interesting in our findings was the existence of significant sex-based differences in UCP4 protein levels in both cortical and hippocampal mitochondria, regardless of the strain. These differences were evident in the cortex throughout the whole time-course examined (from 2 to 14 months), but not in the hippocampus. Lower levels of UCP4 protein in male brains as compared to females are consistent with existing literature [66], and our previous studies with CRND8 mice [60]. Further, lower levels of UCP4 were detected in the cortex and hippocampus of 3xTg female mice, but not males, as early as at 2 months of age. Genetic variants of UCP4 were shown to increase susceptibility to late-onset AD, and the significant decrease that we found in 3xTg female, but not male mice, may represent a vulnerability factor for female brain mitochondria. Chan et al. showed that UCP4 can affect store-operated calcium entry and mitochondrial sequestration of calcium [70]. It was also reported that mutations in UCP4 decrease succinate-driven Complex II respiration [71], and our previous study already showed decreased succinate-driven Complex II respiration in the cortex of 3xTg males [11]. The expression of UCP4 has been found to be significantly reduced in the AD brains. De la Monte et al. [72] found reduced levels of UCP 2, 4, and 5 in the frontal lobe of AD patients. The study of Thangavel et al. [73] showed down-regulation of mitochondrial UCP2 and UCP4 in parahippocampal gyrus of AD brains compared to non-AD brains and argued that this state is responsible for exacerbation of AD pathophysiology. Their observations suggest that either a low mitochondrial uncoupling or proton gradient dissipation might play a role in APP processing.

## 5. Conclusions

Mitochondrial impairment may be an early driver of neuronal degeneration in AD brain and may represent a vulnerability factor for the onset and progression of the disease, particularly in females. Hypometabolism and mitochondrial dysfunction and changes in markers of dynamics show predicted patterns in 3xTg mice but exhibit different rates of alteration depending on sex, brain region, and disease progression. These findings highlight the importance of controlling and accounting for sex, brain region, and age in studies examining brain bioenergetics using this common AD model in order to more accurately evaluate potential therapies and improve the gender-specific translatability of preclinical findings.

## Figures and Tables

**Figure 3:**
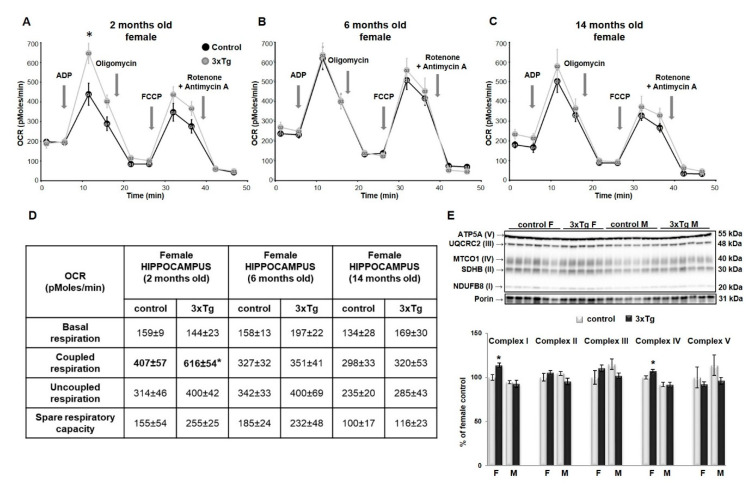
Representative measurements of oxygen consumption rates in mitochondria isolated from the hippocampus of 3xTg and age-matched control females at 2 months (**A**), 6 months (**B**) and 14 months of age (**C**). Coupled respiration (Table **D**, in bold) was significantly increased (* *p* < 0.05) only in 2 months old 3xTg female mice. No changes were detected in 3xTg males at any age. Data are expressed as OCR in pmoles/min, and values are expressed as mean ± SEM of *n* = 4–6 replicates. Representative WB (**E**, **upper** panel) and relative quantification (**E**, **lower** panel) for protein levels of OXPHOS subunits normalized to porin, in hippocampal mitochondria from 3xTg female and male mice and their respective controls at 2 months of age. Results are expressed as percent change from control mean of 100% ± SEM of *n* = 4–6 animals per group (* *p* < 0.05). No changes were detected in protein levels of OXPHOS subunits in hippocampal mitochondria from 3xTg mice at 6 and 14 months (data not shown).
